# Ginsenoside Rg1 protects starving H9c2 cells by dissociation of Bcl-2-Beclin1 complex

**DOI:** 10.1186/s12906-016-1112-2

**Published:** 2016-05-26

**Authors:** Dan Li, Jun Wang, Jincai Hou, Jianhua Fu, Dennis Chang, Alan Bensoussan, Jianxun Liu

**Affiliations:** Institute of Basic Medical Sciences, Xiyuan Hospital, China Academy of Chinese Medical Sciences, Beijing Key Laboratory of Pharmacology of Chinese Materia Medica, Hai Dian District, Beijing, China; Institute of Basic Theory, China Academy of Chinese Medical Sciences, Dong Cheng District, Beijing, China; National Institute of Complementary Medicine, University of Western Sydney, Sydney, Australia

**Keywords:** Ginsenoside Rg1, Autophagy, Apoptosis, Dynamic relationships, Cardiomyocytes

## Abstract

**Background:**

Autophagy can result in cellular adaptation, as well as cell survival or cell death. We investigated how ginsenoside Rg1(G-Rg1) regulates the relationship between autophagy and apoptosis induced by continuous starvation.

**Methods:**

H9c2 cells under continuous starvation were treated with or without ginsenoside Rg1, and autophagy and apoptosis related proteins were assessed over a continuous time course by Western blot. Dynamic fluorescence intensity of green fluorescent protein (GFP)-LC3 was used to assess autophagosome formation by live cell imaging. Cyan fluorescent protein (CFP) -Beclin1(BECN1) and yellow fluorescent protein (YFP) -Bcl-2 were co-transfected into cells to observe ginsenoside Rg1 regulation of BECN1/Bcl-2 interaction using Fluorescence Resonance Energy Transfer (FRET). Immunoprecipitation was also used to assess BECN1/Bcl-2 interaction over a continuous time course.

**Results:**

In H9c2 cells, starvation induced both apoptosis and autophagy. Cell apoptosis was significantly attenuated in ginsenoside Rg1-treated conditions, while autophagy was promoted. Ginsenoside Rg1 weakened the interaction between Beclin1 and Bcl-2, inhibiting apoptosis while promoting autophagy. Our results suggest that autophagy is beneficial to starved cardiac cells over a period of time. Furthermore, we describe the effect of ginsenoside Rg1 on the relationship between autophagy and apoptosis during starvation.

**Conclusions:**

Our findings provide valuable evidence for employing ginsenoside Rg1 as a specific promoter of autophagy and inhibitor of apoptosis.

**Electronic supplementary material:**

The online version of this article (doi:10.1186/s12906-016-1112-2) contains supplementary material, which is available to authorized users.

## Background

Although the concept of autophagy was established decades ago, the exact role it plays in pathological processes remains unclear. In cardiomyocytes, autophagy plays a dual role, demonstrating either protective or harmful effects under different pathological conditions [1–5]. Some studies suggest that autophagy protects cells from oxidative stress and apoptosis, a key survival mechanism, and maintains energy homeostasis and viability in cell starvation models [6–11]. However, other studies suggest that autophagy can enhance cardiomyocyte damage induced by high glucose levels [12] and ischemia/reperfusion [13]. It is somewhat surprising that even under the same pathological condition (e.g. starvation), autophagy can exert both pro-survival and pro-death effects, two seemingly opposing roles. It has been demonstrated that excessive autophagy leads to cell death under starvation conditions. Thus, the extent of autophagy appears to be critical for determining whether it will play a protective or harmful role [14]. However, little research has been done to assess the dynamic process of autophagy during starvation. It is currently unclear if the level of autophagy changes at different time points during starvation and, if so, how this might impact cardiomyocyte health. Therefore, pharmacological effects should be evaluated in a dynamic fashion, rather than relying on a single time point.

Ginsenosides are a group of nontoxic, bioactive components derived from the rhizome of Panax ginseng, one of the mostly commonly used Chinese herbs. Growing evidence suggests that ginsenosides possess protective abilities against coronary artery disease, myocardial ischemia, cardiac hypertrophy, heart failure and arrhythmia [15–20]. Ginsenoside Rg1, one of the main members of the ginsenoside family, has been shown to promote angiogenesis [21–24], to exert anti-hypertension effects [25] and to positively affect myocardial remodeling [26–28]. A recent study indicated that Rg1 inhibits apoptosis by regulating autophagy [13], but the exact mechanism remains unclear. Furthermore, no study to date has assessed the dynamic regulation of autophagy by Rg1.

Autophagy and apoptosis can occur simultaneously in response to starvation, and the interplay between autophagic and apoptotic pathways is emerging as a crucial process in determining the initiation of programmed cell death [29, 30]. Beclin 1, a Bcl-2 interacting protein, has been implicated as an important regulator of autophagy [31]. Indeed, the suppression of Beclin 1 expression impairs autophagy and sensitizes the cells to starvation-induced apoptosis [32]. Beclin 1 interacts with anti-apoptotic multi-domain proteins of the Bcl-2 family, in particular Bcl-2 and its homologue Bcl-X(L), by virtue of its BH3 domain, an amphipathic alpha-helix that binds to the hydrophobic cleft of Bcl-2/Bcl-X(L) [33, 34]. It has been shown that the interaction between Bcl-2 and Beclin1 leads to the inhibition of autophagy by interfering with the formation and activity of the autophagy promoter complex, Beclin 1/hVps34 [35]. It has not yet been determined whether ginsenoside Rg1 regulates autophagy through Bcl-2 and Beclin1.

In the present study, we sought to address the dynamic regulation of ginsenoside Rg1 on autophagy for protecting cardiomyocytes from apoptosis under prolonged starvation and the related molecular mechanism by which ginsenoside Rg1 regulates autophagic activity.

## Methods

### Materials

Commercial antibodies and chemicals were purchased from the following sources: anti-LC3 (L7543) and anti-caspase 3 (C8487) antibodies, rapamycin (Rap;R8781) and 3-methyladenine (3-MA;M9281) were purchased from Sigma-Aldrich; anti-Beclin1 (3738s), anti-Bcl-2 (2870s) and anti-GAPDH (5174s) antibodies were purchased from Cell Signaling Technology. Ginsenoside Rg1 (Purity > 95 %, 110703–201027) were chemically standardized products obtained from the National Institutes for Food and Drug Control, which was validated by fingerprint chromatographic methodologies.

### Starvation model in H9c2 cells and drug treatment

The rat ventricular cell line H9c2 was purchased from the cell bank of the Chinese Academy of Medical Sciences (Beijing, China). Cells were cultured in Dulbecco’s modified Eagles medium (DMEM, Hyclone) containing 10 % fetal bovine serum (GIBCO), 40 U/ml penicillin and 40 U/ml streptomycin in a humidified atmosphere of 95 % air/5 % CO_2_ at 37 °C. Cells were grown to 80 % confluency before being exposed to starvation. To create starvation conditions, cardiac myocytes were washed three times with phosphate-buffered saline (PBS) and were incubated in glucose-free, serum-free DMEM (GIBCO, 11966) at 37 °C for 0, 30, 60, 90, 120, 150, 180, 210 or 240 min or until cell death. Cells cultured in nutrient-rich medium were used as a control. For the active treatment study, cells were pre-treated with ginsenoside Rg1 (Rg1) for 12 h in nutrient-rich medium and then were treated with ginsenoside Rg1 for 0, 30, 60, 90, 120, 150, 180, 210 or 240 min or until cell death in glucose-free, serum-free DMEM. Rg1 was first dissolved in DMSO and then diluted to the final concentration in culture medium (final DMSO concentration <1 %).

### Cell viability assay

Cells were seeded in 96-well plates (2 × 10^4^ cells per well). After 48 h, cell viability was measured using the 3-(4,5)-dimethylthiahiazo (−z-y1)-3,5-diphenytetrazoliumromide (MTT) assay at different time points (30, 60, 90, 120, 150, 180, 210 or 240 min) following glucose-deprivation. MTT was dissolved and sterilized in PBS at 0.5 mg/ml, and 100 μL of this solution was added to each well. The plate was incubated at 37 °C for 4 h before the media was removed. Approximately 200 μL of dimethyl sulfoxide (DMSO) was added into each well, and the plate was gently rocked for 10 min to dissolve the dark blue MTT crystals. Cell viability was expressed as optical density (OD), which was detected by use of a microplate reader at 490 nm. The experimental groups included one normal control group (starvation for 0 min), eight starvation model groups (starvation for 30, 60, 90, 120, 150, 180, 210 or 240 min) and twenty-seven ginsenoside Rg1 treatment groups (treated with 25, 50 or 100 μM ginsenoside Rg1 for each time point).

### Live cell imaging system for green fluorescent protein (GFP)-LC3

Live cell imaging experiments were carried out for one model group and one drug treatment group. The treatment group was treated with 100 μM ginsenoside Rg1, as described above. The H9c2 cells were transfected with GFP-LC3 expression plasmids using an electroporation apparatus (BTX). The electroporation parameters used were as follows: square-wave, 100 V/cm, 0.5 ms and 5 impulses. Electroporation was carried out in low conductivity electroporation buffer (10 mM phosphate, 1 mM MgCl_2_, 250 mM sucrose, pH 7.4). Cells were allowed to recover for 20 min following electroporation and before being placed in normal medium and returned to normal culture conditions. After 48 h, the transfected cells were glucose deprived for 24 h. The dynamic changes in color and fluorescence intensity were evaluated in the same cell selected at different times using the live cell imaging system (Olympus).

### Terminal dexynucleotidyl transferase-mediated dUTP nick end labeling (TUNEL) assay

TUNEL experiments were carried out for nine model and nine treatment groups. The treatment groups were treated with 100 μM ginsenoside Rg1, as described above. TUNEL assays (Roche, 1684795) were then performed according to the protocol provided by the manufacturer. Slides rinsed with PBS were counterstained with 1 mM of Hoechst stain (Sigma, 861405). The fluorescein isothiocyanate (FITC)-labeled TUNEL-positive cells were imaged using the Synergy™ 4 Hybrid Microplate Reader system (BioTek) with 488 nm excitation and 530 nm emission wavelengths. The cells with green fluorescence were classified as apoptotic cells.

### Flow cytometric analysis

Flow cytometry was carried out for nine model and nine treatment groups (allocated as in the TUNEL assay). H9c2 cells with or without Rg1 treatments were trypsinized and resuspended in cell culture medium; at least 10^5^ cells were collected. The cells were washed twice with PBS, then 5 μl FITC-Annexin V and 5 μl propidium iodide (PI) were added to the cells, and the cells were incubated in the dark at room temperature for 15 min. Binding buffer was added, and cells were analyzed by flow cytometry within 30 min.

### Immunoprecipitation assay

A time-effect study was carried out for nine model and nine treatment groups (allocated as in the TUNEL assay). Whole-cell lysates were used for immunoprecipitation with antibodies against Beclin1. Ten micrograms of antibody was incubated with 1 ml of cell lysate at 4 °C overnight. After the addition of Protein A/G-agarose beads, the incubation was continued for a further 4 h. After centrifugation, the immunoprecipitates were extensively washed with lysis buffer 3–4 times and eluted with loading buffer. Bcl-2 antibodies were used in the subsequent Western blot experiments.

### Western blot analysis

Western blot experiments were carried out for nine model and nine treatment groups (allocated as in the TUNEL assay). Protein samples were extracted from H9c2 cells. Preparation of protein samples consisted of several steps, including splitting, centrifugation and boiling. Protein samples (20 μg) were fractionated by sodium salt (SDS)-Polyacrylamide gel electrophoresis (PAGE) (10 or 12 % polyacrylamide gels), transferred to a polyvinylidene difluoride (PVDF) membrane and then blocked in 5 % bovine serum albumin and prepared in a Tris-buffered saline (TBS) for 1 h at room temperature. The membranes were incubated with primary antibodies (GAPDH as a loading control) overnight at 4 °C then washed five times with 0.1 % Tween-20 in Tris-buffered saline (TBS-T) for 30 min each. The membrane was then incubated with horseradish peroxidase (HRP)-conjugated secondary antibodies for 1 h at room temperature. After washing, the immunoreactive protein bands were developed by enhanced chemiluminescence (ECL), and the resulting membranes were imaged using the gel imaging system (BIO-RAD).

### FRET measurements

FRET experiments were carried out for five groups, including one control group, one model group and three treatment groups (25, 50 and 100 μM ginsenoside Rg1 pretreatment, respectively). Experiments were performed in H9c2 cells stably co-expressing Cyan fluorescent protein (CFP) -BECN1 and Yellow fluorescent protein (YFP) -Bcl-2. The cells were washed with glucose-free Tyrode’s solution three times and cultured in glucose-free Tyrode’s solution with or without Rg1 treatment at 37 °C until cell death. Fluorescence was measured with the microplate system every 10 min with the excitation wavelength of CFP and emission wavelength of YFP set at 436 nm and 527 nm, respectively.

### Statistical analysis

Quantitative data were expressed as mean ± standard deviation (SD). Differences between experimental groups were examined by one-way analysis of variance (ANOVA), and means of two groups were compared using Student’s *t*-test (paired, 2-tailed) by SPSS 18.0 software. For all analyses, *p* <0.05 was considered statistically significant.

## Results

### Ginsenoside Rg1 inhibits starvation induced-apoptosis, contributing to cardioprotection

**Fig. 1:**
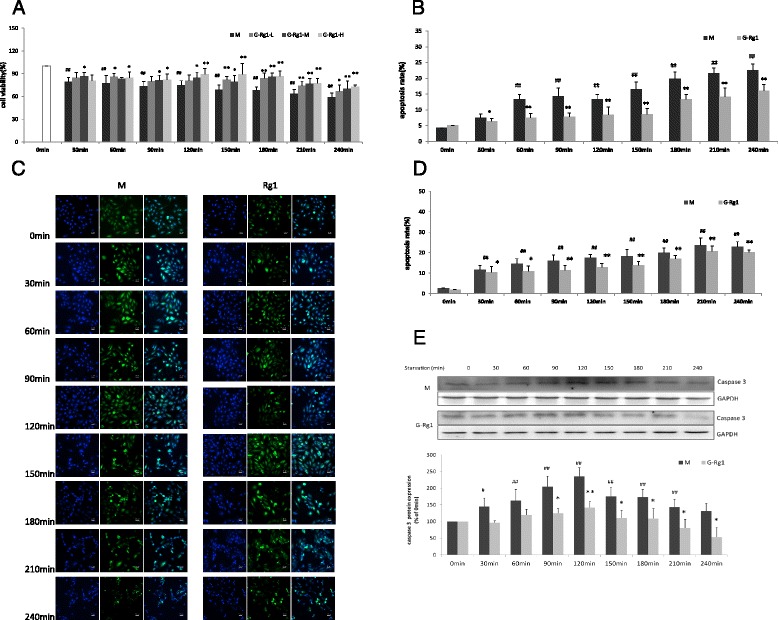
The protective effects of ginsenoside Rg1 on starvation-induced apoptosis in H9c2 cells. **a** Effects of ginsenoside Rg1 doses on the cell viability of H9c2 under starvation at 0, 30, 60, 90, 120, 150, 180, 210 and 240 min. H9c2 cells were incubated with ginsenoside Rg1 at either 25 (G-Rg1-L), 50 (G-Rg1-M) and 100 μM (G-Rg1-H) for 12 h. The cells were then cultured with or without ginsenoside Rg1 in serum and glucose-free DMEM over 240 min. The time-course of cell viability was determined by MTT assay. Values are expressed as the mean ± SD, *n* = 3. ^#^ *p* < 0.05, ^##^ *p* < 0.01, starvation model group (M) versus control group (0 min); *p < 0.05, **p < 0.01, ginsenoside Rg1 treatment groups (G-Rg1-L, G-Rg1-M, G-Rg1-H) compared with the corresponding time point of starvation model (M). **b**, **c** Effects of ginsenoside Rg 1 on starvation-induced apoptosis in H9c2 cells using TUNEL and Hoechst staining. **d** Effects ofginsenoside Rg1 on starvation-induced apoptosis in H9c2 cells using flow cytometric analysis (Additional file 1: Figure S2). **e** Effects of ginsenoside Rg1 on expression of caspase 3 in H9c2 cells using Western blot analysis. Densitometric analysis was used to quantify the levels of active caspase 3 in H9c2 cells with and without ginsenoside Rg1 treatment. Data is shown as mean ± SD, *n* = 3. *p < 0.05, **p < 0.01, starvation model group (30–240 min) versus the corresponding time point of starvation model group

During starvation, cell survival significantly decreased in a time-dependent manner between 30 and 240 min when compared with the 0 min (Additional file 1: Figure S1). At 240 min, 40 % cell death had occurred, so this time point was deemed an adequate duration for serum and glucose deprivation in subsequent experiments. To evaluate whether Rg1 protects H9c2 cardiomyocytes under starvation conditions, cell viability was also measured after pretreatment with cultured media containing Rg1 at all three concentrations (100, 50, 25 μM). Cell viability significantly increased at 120, 150, 180, 210 and 240 min with Rg1 treatment (Fig. 1a). Moreover, a dose-dependent effect was also observed between 120 and 240 min (Fig. 1a). Consequently, the higher, more effective dose of ginsenoside Rg1 (100 μM) was chosen for all subsequent experiments.

Annexin V-FITC flow cytometric analysis showed that the population of Annexin V positive/PI negative cells increased in a time-dependent manner. Rg1 treatment decreased apoptosis by 5 to 15 % between 60 and 240 min when compared to the corresponding time point of starvation model group (*P* < 0.05 or *P* < 0.01, Fig. 1b). Similarly, TUNEL assay results showed that Rg1 treatment reduced the starvation-induced increase of TUNEL-positive cells at all time points (Fig. 1c, d).

A time-dependent increase in active caspase 3 levels was observed between 0 and 120 min followed by a time-dependent decline in both the model and Rg1 groups. In both cases, peak levels were observed at 120 min after induction of starvation. Rg1 treatment significantly decreased the levels of active caspase 3 by 39, 40, 37, 37, 44 and 59 % at 90, 120, 150, 180, 210 and 240min after induction of starvation respectively when compared to the model group (Fig. 1e).

### Rg1 promotes starvation-induced autophagy while inhibiting apoptosis in H9c2 cells

**Fig. 2:**
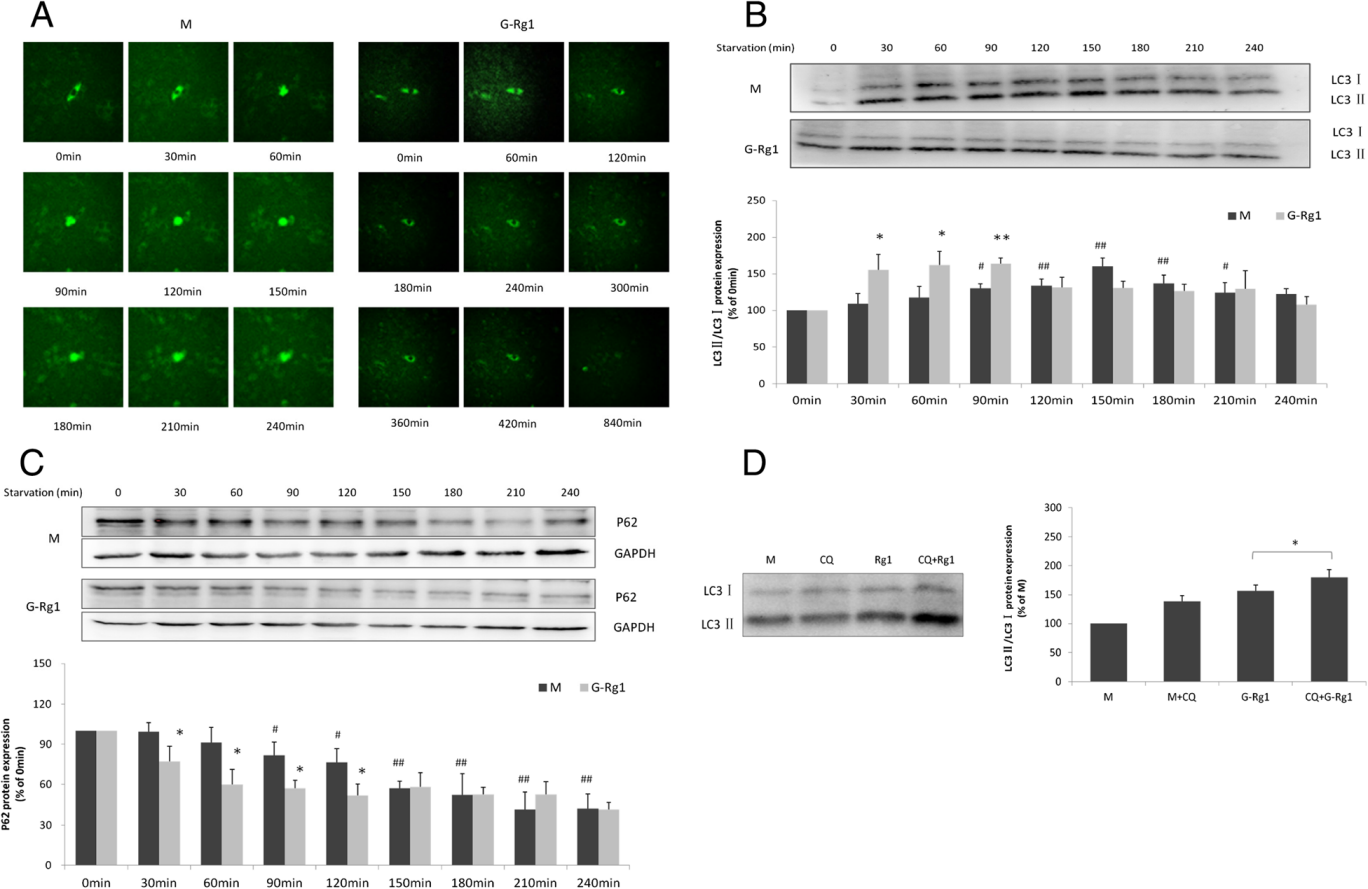
Ginsenoside Rg1 induces autophagy in H9c2 cells under starvation conditions. **a** Representative live cell images of GFP in H9c2 cells transfected with GFP-LC3 for 48 h in the presence or absence of ginsenoside Rg1 under starvation. **b**, **c** Western blot analysis of endogenous LC3 and p62 in H9c2 cells after ginsenoside Rg1 treatment. Densitometric analysis was used to quantify the levels of LC3-II and p62 in H9c2 cells with and without ginsenoside Rg1 treatment. Values are expressed as the mean ± SD, n = 3, ^#^ *p* < 0.05, ^##^ *p* < 0.01, starvation model group (30–240 min) versus starvation 0 min model group or *p < 0.05, ginsenoside Rg1 treatment group versus the corresponding time point of starvation. Trend changes of LC3-II and p62 in H9c2 cells with and without ginsenoside Rg1 treatment. **d** Effects of ginsenoside Rg1 on LC3 levels in H9c2 cells in the presence or absence of 5 μM CQ at 90 min after starvation by Western blot analysis. *p < 0.05, H9c2 cells in the presence of CQ and ginsenoside Rg1 group (CQ + G-Rg1) versus ginsenoside Rg1-only group (G-Rg1)

In the starvation model group, green fluorescence intensity persistently increased over 90 min accompanied by contraction of the cells. Four hours (240 min) after the induction of starvation, the cells contracted to a spherical shape, their movement stopped and the fluorescence was quenched. In the Rg1 treatment group, however, green fluorescence intensity began to increase at starvation 60 min,then fluorescence was stable until quenched after starvation 840 min and the normal cell morphology was maintained over 420min after starvation (Fig. 2a), suggesting that autophagic vacuolization may have occurred and cell death was delayed in response to ginsenoside Rg1 treatment.

As inefficient autophagic vesicle turnover (autophagosome-lysosome fusion and/or down-stream cargo degradation) can lead to autophagic vesicle accumulation [33], we proposed that Rg1 might induce autophagic vacuolization by impairing autophagic vesicle turnover. Therefore, protein levels of LC3 and p62 in H9C2 cells were assessed under starvation conditions at different time point following induction of starvation. Western blot analysis revealed that the LC3-II/LC3-I ratio was progressively up-regulated during starvation, achieving statistical significance at 90 min and peaking at 150 min, before starting to decline (Fig. 2b), while p62 protein levels were progressively reduced under starvation (Fig. 2c). When compared with the corresponding time point of starvation model group, ginsenoside Rg1 treatment significantly increased the LC3-II/LC3-I ratio by 42, 37, 25 % at 30, 60 and 90 min respectively (*P* < 0.05,or *P* < 0.01) and decreased p62 protein levels at 30, 60, 90 and 120 min (*P* < 0.05). These results suggest that Rg1 promotes autophagic activity in parallel with the inhibition of apoptosis in H9c2 cells.

As illustrated in Fig. 2d, the expression of LC3-II was significantly elevated in the presence of both chloroquine(CQ) and Rg1 when compared to the model, CQ and ginsenoside Rg1 treatment-only groups. This suggests a CQ-induced accumulation of autophagic vesicles induced by Rg1 treatment in H9c2 cells under starvation. This result provides further evidence to support the concept that Rg1 promotes autophagy, without however significantly impairing autophagic vesicle turnover. (The effect of CQ on differet time pionts showed in Additional file 2: Figure S3).

### Rg1 mediates BECN1-Bcl- 2 interactions in H9c2

**Fig. 3:**
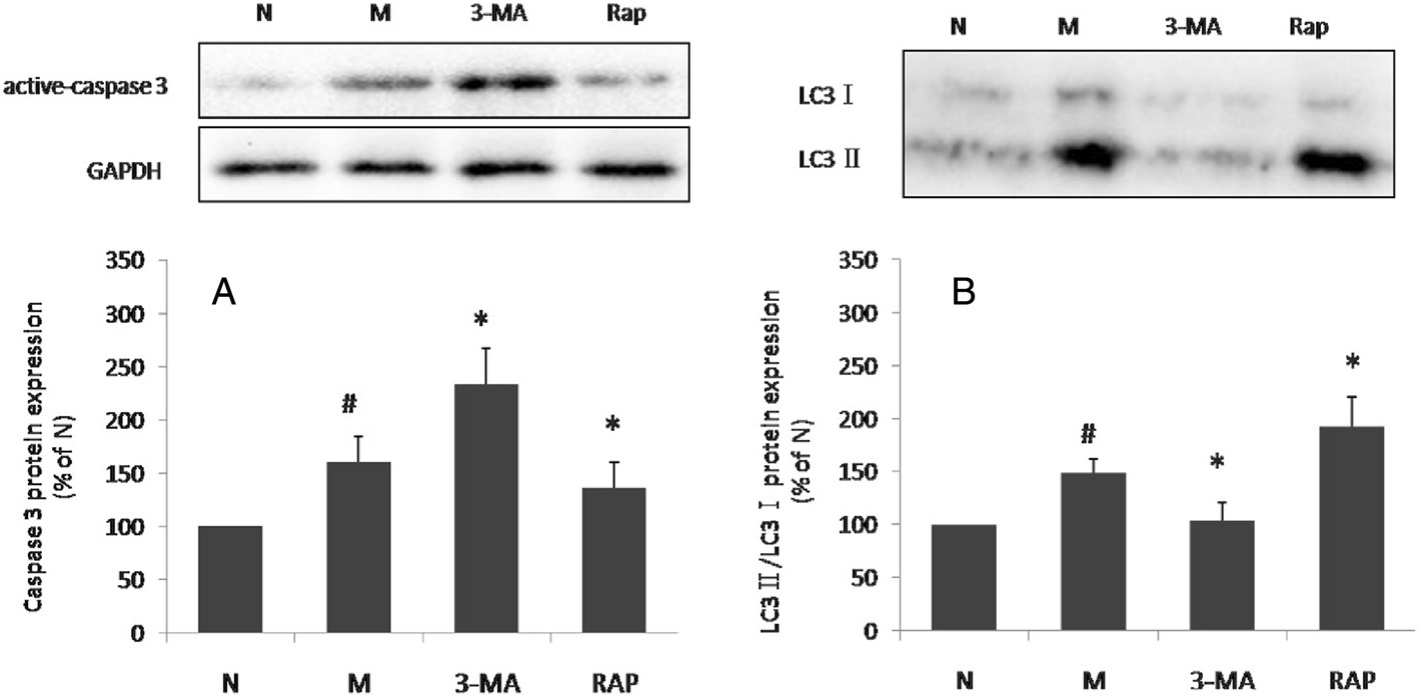
Effects of 3-MA Rap on LC3 and active caspase 3 in H9c2 cells. H9c2 cells were treated with 3-MA for 24 h or Rap for 12 h under normal conditions and for 150 min under starvation. Expression of LC3 and active caspase 3 was determined by Western blot. Values are expressed as the mean ± SD, *n* = 3. ^#^ *p* < 0.05 model group versus normal group; *p < 0.05, ginsenoside Rg1 treatment group verse starvation model group (The effect of 3-MA and Rap on different time points showed in Additional file 2: Figures S4 and S5)

Caspase 3 levels (Fig. 3a) increased in the presence of the inhibitor 3-MA and decreased in the presence of Rap at the same time point. 3-MA and Rap could inhibit or increase the expression of LC3-II of the model cell (Fig. 3b). The combined data suggested that promoting autophagy could suppress apoptosis and may be one of the mechanisms underlying the protective effect of Rg1 on starving H9c2 cells. This result also highlights a potential therapeutic target for reducing starvation-induced apoptosis via promoting autophagy.

**Fig. 4:**
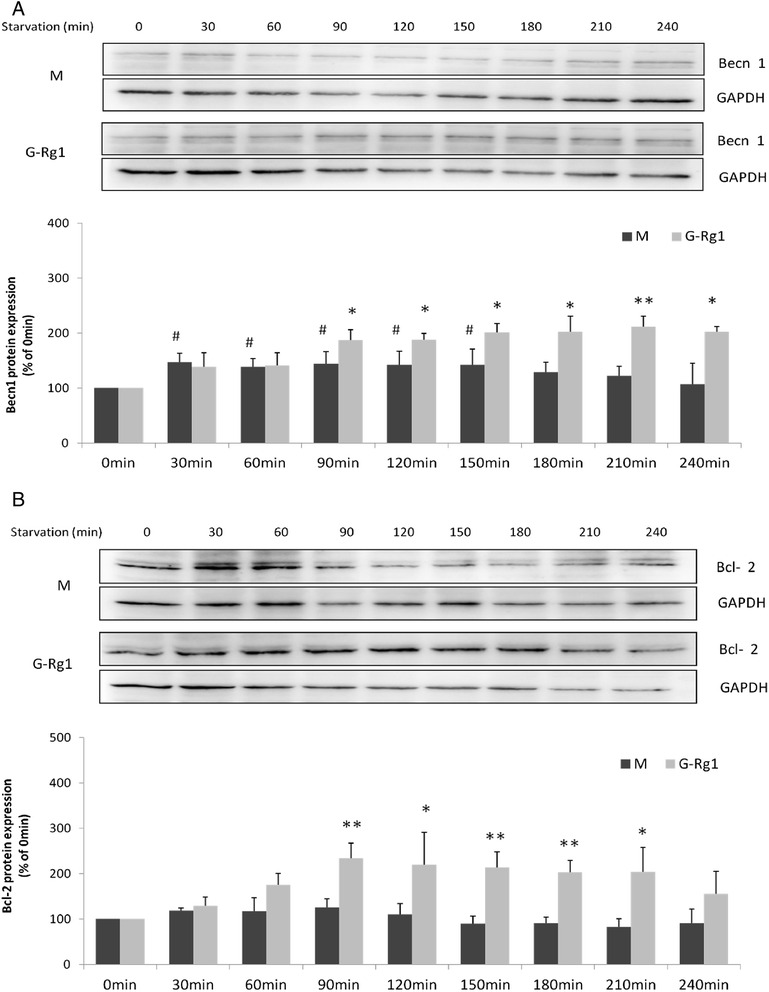
Increased expression of Beclin 1 and Bcl-2 in response to ginsenoside Rg1 in H9c2 cells under starvation conditions. **a**, **b** Western blot analysis of endogenous Beclin 1 and Bcl-2 in H9c2 cells after treatment with and without ginsenoside Rg1. Values are expressed as the mean ± SD, *n* = 3, ^#^ *p* < 0.05, ^##^ *p* < 0.01, starvation model group (M) verse control group (0 min) or *p < 0.05, **p < 0.01, tarvation 30-240min model group verse starvation 0min model group or *p < 0.05, **p < 0.01, ginsenos ide Rg1 treatment group (G-Rg1) verse the corresponding time point of starvation model group

Beclin1 expression was upregulated between 30 and 150 min after commencement of starvation and then began to decline to a statistically insignificant level (Fig. 4a). Meanwhile, no significant changes in Bcl-2 expression were noted in response to starvation. Treatment with Rg1 markedly increased levels of Beclin-1 and Bcl-2 at most time points. When compared to the corresponding time of starvation model group, Rg1 treatment significantly increased Beclin-1 protein levels by 24 to 88 % between 90 and 240 min (Fig. 4a) and Bcl-2 protein levels by 87 to 147 % between 90 and 210 min (Fig. 4b).

**Fig. 5:**
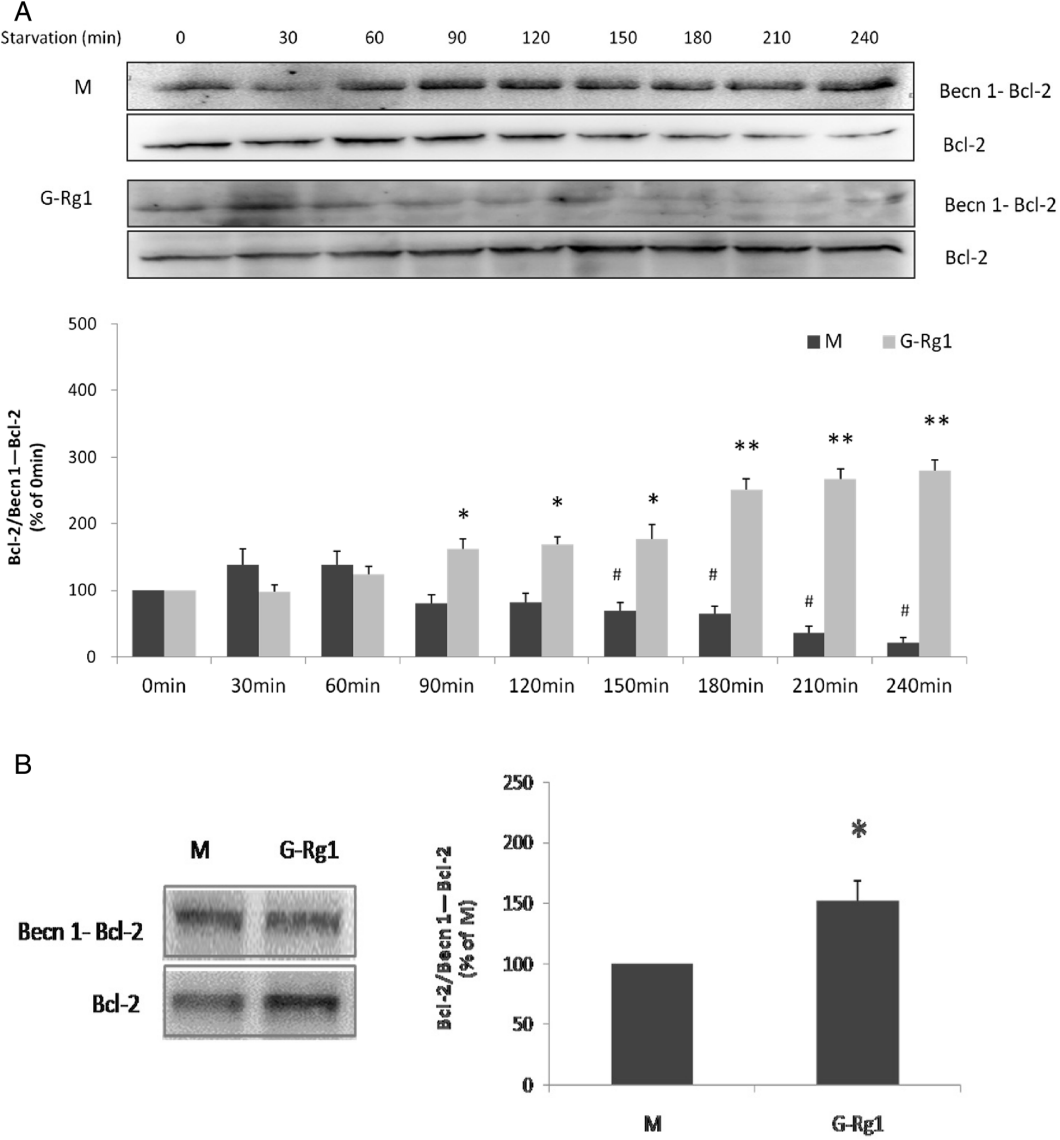
Effects of ginsenoside Rg1 on the interaction of Beclin 1 and Bcl-2 in H9c2 cells by immunoprecipitation. **a**, **b** Immunoprecipitation of Beclin 1 and Bcl-2 with or without ginsenoside Rg1 treatment demonstrated time-dependent changes (**a**) and the effect of 100 μM Rg1(**b**). Cell lysate was extracted for immunoprecipitation with anti-Bcl-2 followed by probing with anti-Beclin1. Values are expressed as the mean ± SD, *n* = 3. ^#^ *p* < 0.05, starvation 30-240min model group verse starvation 0min model group or *p < 0.05, ginsenoside Rg1 treatment group verse the corresponding time point of starvation model group

The immunoprecipitation and Western blot analyses showed that BECN1 levels immunoprecipitating with Bcl-2 steadily increased in a time-dependent manner during starvation while free Bcl-2 levels decreased gradually. Conversely, Rg1 treatment upregulated Bcl-2 while weakening the BECN1-Bcl-2 interaction in a time-dependent manner (Fig. 5a). One hundred micrometer Rg1 is the optimal dosage for protecting H9C2 cells from starvation. It upregulated the ratio of Bcl-2 and BECN1-Bcl-2 by 35 % (Fig. 5b).

**Fig. 6:**
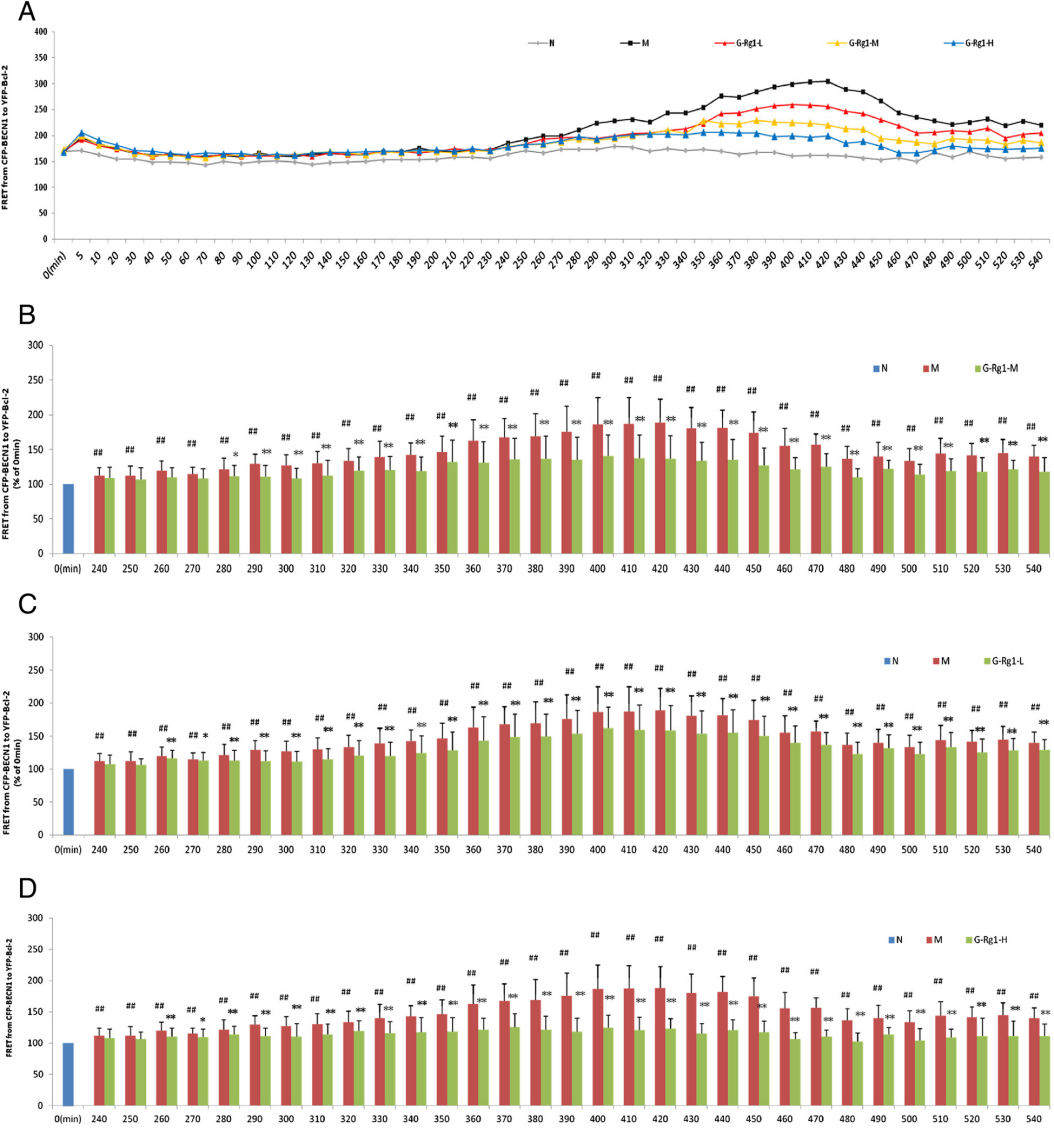
Effects of ginsenoside Rg1 on the interaction of Beclin 1 and Bcl-2 in H9c2 cells by FRET. **a** Effect of ginsenoside Rg1 at low (**b**, 25 μM), medium (**c**, 50 μM) and high (**d**, 100 μM) doses on FRET from CFP-Beclin 1 to YFP-Bcl-2 in live H9c2 cells. Compared to the corresponding time point of starvation model group, ginsenoside Rg1 treatment decreased FRET in live cells from 240 to 470 min in a dose-dependent manner. Values are expressed as the mean ± SD, *n* = 3. ^##^ *p* < 0.01, starvation 240–480 min model group (M) verse starvation 0 min model group or *p < 0.05, **p < 0.01, ginsenoside Rg1 treatment group verse the corresponding time point of starvation model group

In the starvation model group, YFP intensity started to increase at 240 min after starvation then peaked at 420 min and commenced a gradual decline, reaching a plateau at 480 min after starvation (Fig. 6a). Treatment with low does of Rg1 (25μM), the YFP intensity markedly decreased from 280 to 540 min when compared to the corresponding time point of model group (Fig. 6b). Respectively, after middle (50μM) and high (100μM) does of Rg1 treatment, YFP intensity began to decrease at starvation 260 and 270 min (Fig. 6c, d). These results suggest that Rg1 produced similar dose-dependent changes in YFP intensity and the interaction between BECN1 and Bcl-2 may have been weakened by ginsenoside Rg1.

## Discussion

Previous studies have revealed multiple pharmacological effects of ginsenoside Rg1. The present study reports for the first time the dynamic regulatory effects of Rg1 on autophagy and the interactions between autophagy and apoptosis in H9c2 cells under starvation. These data provide evidence that Rg1 can partially inhibit the process of apoptosis by enhancing autophagy under continuous starvation conditions.

Autophagy is an evolutionarily conserved pathway that involves the sequestration and delivery of cytoplasmic materials to the lysosomes, where proteins are degraded and recycled [36]. It is involved in organelle turnover and bioenergetic management during starvation. In recent years, the regulation and contribution of the autophagic process to cell metabolism has been characterized in great detail. As a process of consuming cellular components to generate energy, autophagy engages in a complex interconnection with apoptosis according to the nature of the stimulus and cell type. It either suppresses apoptosis by eliminating damaged organelles under cellular stress or sensitizes cells to apoptosis, acting as an energy source. There remains considerable debate as to the relationship between autophagy and apoptosis. The dynamic processes of autophagy and apoptosis have prompted the need to understand how autophagy and apoptosis interact at different stages and in response to various conditions.

In the starvation model in H9c2 cells described here, marker proteins of autophagy were initially evaluated. The conversion of LC3-I (18 kDa) to LC3-II (16 kDa) is a strong biochemical marker for the induction of autophagy [37, 38]. The levels of LC3-II at different time points under starvation condition were thus measured. The LC3-II/LC3-I ratio was significantly increased at 90, 120, 150, 180 and 210 min after starvation when compared to the control (Fig. 2b). This result is consistent with that of Han et al. [37] who reported that the accumulation of LC3-II in primary cardiomyocytes increased at 1, 2, 3 and 6 h under starvation conditions. The existence of an induced autophagic flux was also evaluated through the analysis of p62 levels, also called sequestosome 1 (SQSTM1). SQSTM1 is an ubiquitin-binding scaffold protein that is localized to the autophagosome via LC3-interaction and is specifically degraded by the autophagy-lysosome system [39]. p62 levels are thus inversely related to autophagic activity. The results of the present study showed that p62 levels were significantly decreased in response to starvation (Fig. 2c).

Apoptosis is the principal mechanism by which cells are physiologically eliminated in metazoan organisms [30]. In the present study, the protein expression levels of caspase 3, an indicator of apoptotic activity, were measured. These results revealed that apoptosis and autophagy occur simultaneously in H9c2 cardiomyocytes under starvation conditions, but the dynamics of each process differ. For example, when the starvation-induced increases in LC3 expression reached their maximum, the caspase 3 levels began to decline (Fig. 1d, e). Significant changes in caspase 3 activity in cells incubated with 3-MA or Rap were observed. In previous studies [12], 2mM 3-MA was shown to effectively block autophagic activity, while 50 nM Rap effectively promoted autophagic activity in cardiomyocytes. The results of the present study showed that 3-MA enhanced starvation-induced caspase 3 activation, whereas Rap restrained its elevation, thus suggesting that autophagy promotion played a protective role in H9c2 cardiomyocytes under starvation conditions (Fig. 3).

Rg1 has been demonstrated to have anti-ischemic, anti-hypoxic and pro-autophagic properties [13]. In this study, the effect of Rg1 on autophagy in H9c2 cardiomyocytes was first investigated through determining the fluorescence intensity of the fusion protein GFP-LC3, which primarily represents the abundance of autophagosomes [40]. To date, few studies have reported the use of a real-time monitoring method for collection of the fluorescent signal. In fact, the real-time monitoring of green fluorescence in a single cell could provide vital information on the complete picture of GFP-LC3 changes in response to starvation over time. In the present study, Rg1 treatment not only increased fluorescence intensity but also delayed the time for fluorescence quenching by several hours. The existence of an induced autophagic flux by Rg1 was also evaluated through the analysis of LC3-II and p62 protein levels. Similarly, the results showed that Rg1 brought forward the high peak time of LC3-II/LC3-I and low peak time of p62, suggesting that Rg1 treatment promotes starvation-induced autophagic activities (Fig. 4). In addition, the effects of ginsenoside Rg1 on apoptosis were also evaluated over multiple time points. As measured by flow cytometry, Western blot and TUNEL assay, Rg1 effectively inhibited starvation-induced apoptosis in H9c2 cardiomyocytes at multiple time points.

To determine the mechanism underlying the regulatory effects of Rg1 on the relationship of autophagy and apoptosis in starving H9c2 cells, the interaction of Bcl2 and BECN1 was investigated as an important link between apoptotic and autophagic activities. The BECN1 protein binds to Bcl2 through its BH3 domain. Under normal circumstances, the BH3-only domain of BECN1 may competitively disrupt the bringing of pro-apoptotic proteins to Bcl-2 or Bcl-XL, thus preventing the induction of apoptosis [41]. Under stress conditions, Beclin 1, which is normally sequestered by Bcl-2, is released to induce autophagy. The results of the present study showed that Rg1 treatment upregulated the levels of Beclin 1 and Bcl-2 protein compared with the starvation model group at a slightly different time point. Immunoprecipitation is a classic method for studies of protein interactions. In the present study, Rg1 promoted the dissociation of BECN1 and Bcl-2, increasing free BECN1 and Bcl-2 in the cytoplasm and consequently triggering the autophagic process in a dose- and time-dependent manner. FRET is a physical phenomenon by which energy from a donor fluorophore in an excited state is non-radiatively transferred to a neighboring acceptor fluorophore through dipole-dipole interactions. The strong dependence of FRET on distance enables the use of FRET to measure molecular interactions at a distance of 1–10 nm. The development of GFP and variants such as yellow fluorescent protein (YFP, 516_Ex_/527_Em_) and cyan fluorescent protein (CFP, 436_Ex_/476_Em_) enables CFP/YFP to be used as a FRET pair. One of the most important findings of this study is that, by using the FRET assay, direct efficiency is provided that ginsenoside Rg1 treatment weakened the interaction between Bcl-2 and Beclin1 in the starving H9c2 cardiomyocytes (Fig. 6).

## Conclusions

Our results support the theory that starvation contributes to the induction of autophagic activities in H9c2 cardiomyocytes. Ginsenoside Rg1 could prevent cellular apoptosis via initiating an autophagic survival response, during which time Rg1 could promote the expression of Beclin1 and Bcl-2 and weaken the interaction between Beclin1 and Bcl-2. This study has demonstrated for the first time a novel role for ginsenoside Rg1 in inducing a beneficial autophagic activity in cardiomyocytes under starvation.

## Additional files

Additional file 1: Figure S1. Cell viability of H9c2 cells. Cell viability significantly decreased in a time-dependent manner between 30 and 240 min when compared with the control (0 min). Values are expressed as the mean ± SD, *n* = 3. ^**^ *p* < 0.01, starvation model group verse control group. **Figure S2.** Effects of ginsenoside Rg1 on starvation-induced apoptosis in H9c2 cells using flow cytometric analysis. (PDF 346 kb) Additional file 2: Figure S3. The effect of CQ on different time points. The LC3 level significantly increased at 90 min (**p* < 0.01). **Figure S4.** The effect of 3-MA on different time points. The LC3 level significantly decreased at 12 h (**p* < 0.05) and 24 h (**p* < 0.05). **Figure S5.** The effect of Rap on different time points. The LC3 level significantly increased at 12 h (***p* < 0.01) and 24 h (**p* < 0.05). (XLSX 33 kb)

## Abbreviations

3-MA: 3-methyladenine
BECN1: Beclin1
CFP: cyan fluorescent protein
CQ: chloroquine
FITC: fluorescein isothiocyanate
FRET: fluorescence resonance energy transfer
GFP: green fluorescent protein
G-Rg1: ginsenoside Rg1
Rap: rapamycin
YFP: yellow fluorescent protein
