# Supplementary figures and images for: Ginsenoside Rg1 protects starving H9c2 cells by dissociation of Bcl-2-Beclin1 complex
Source: BMC Complement Altern Med. 2016 May 26;16:146. doi: 10.1186/s12906-016-1112-2 (PMC4881172; doi:10.1186/s12906-016-1112-2)

Fig. S1

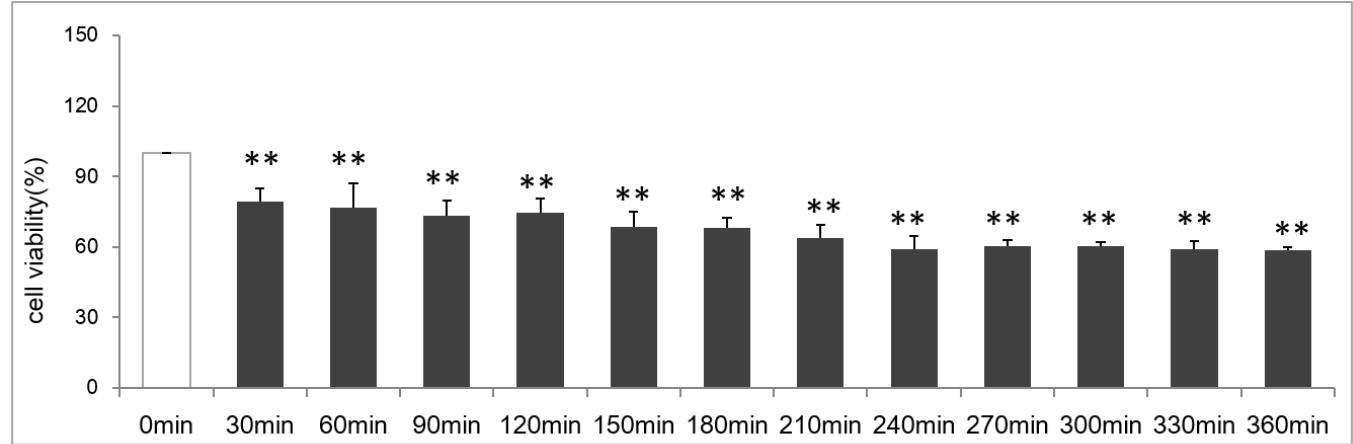

Fig.S2

M

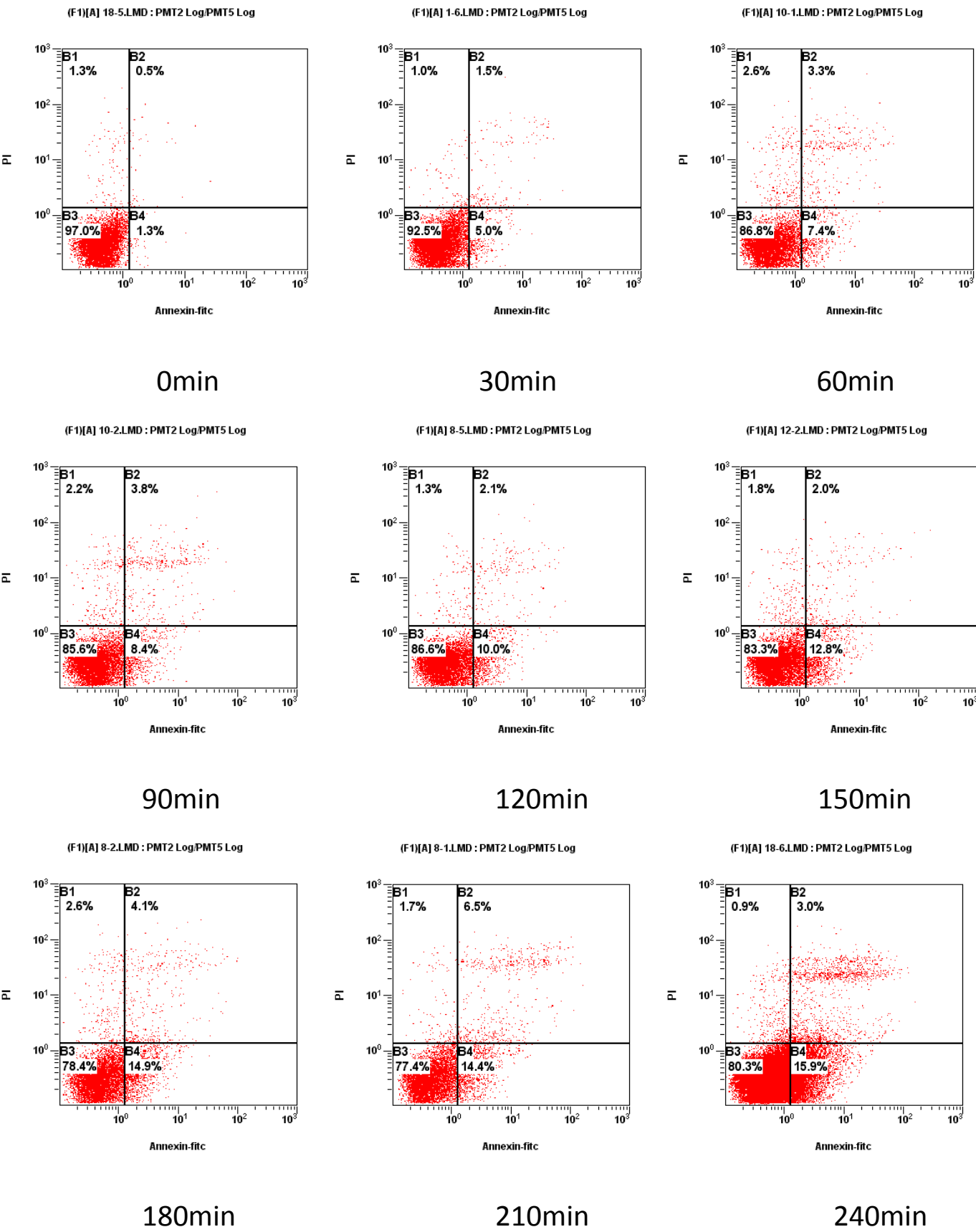

# Rg1

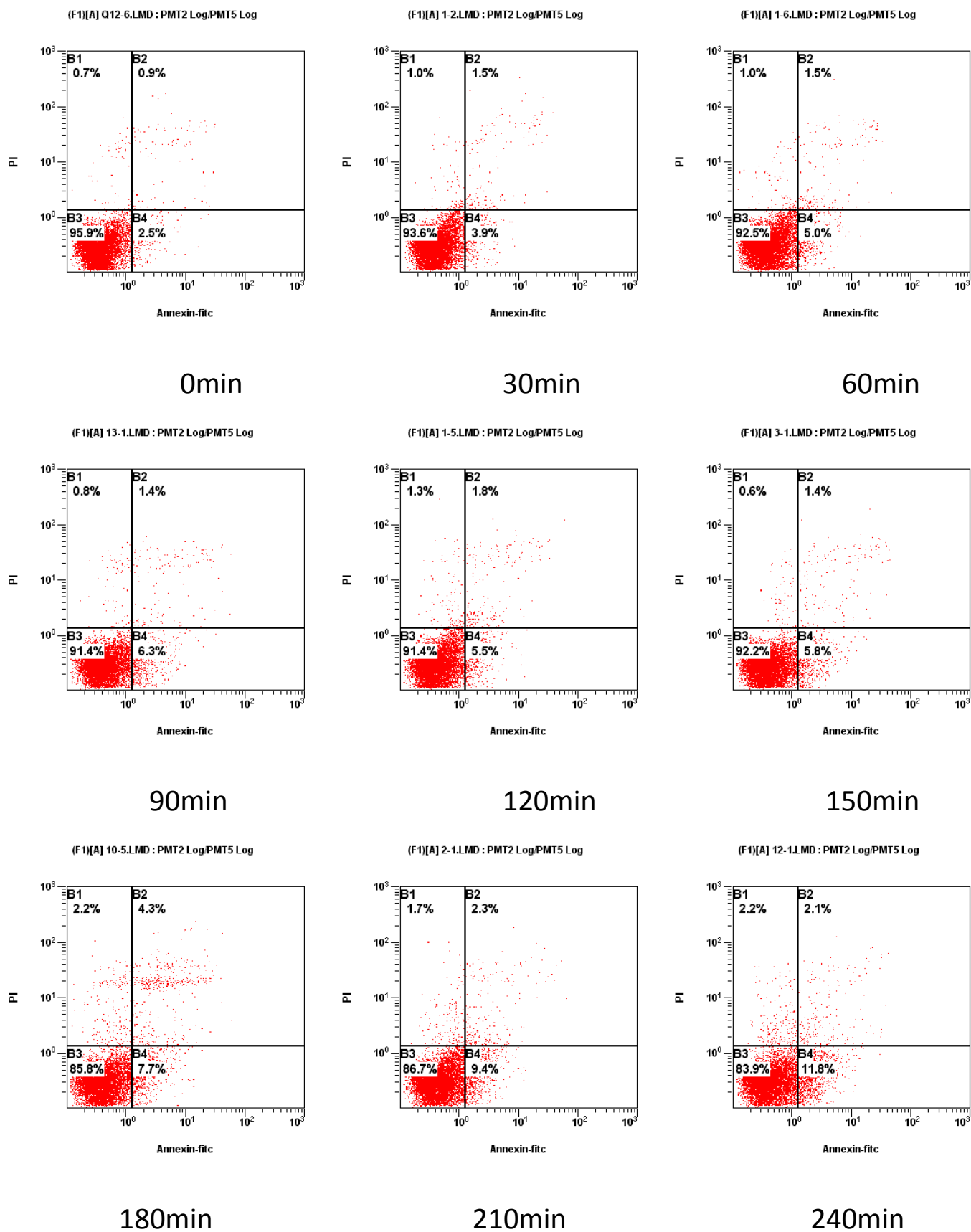

Fig. S3

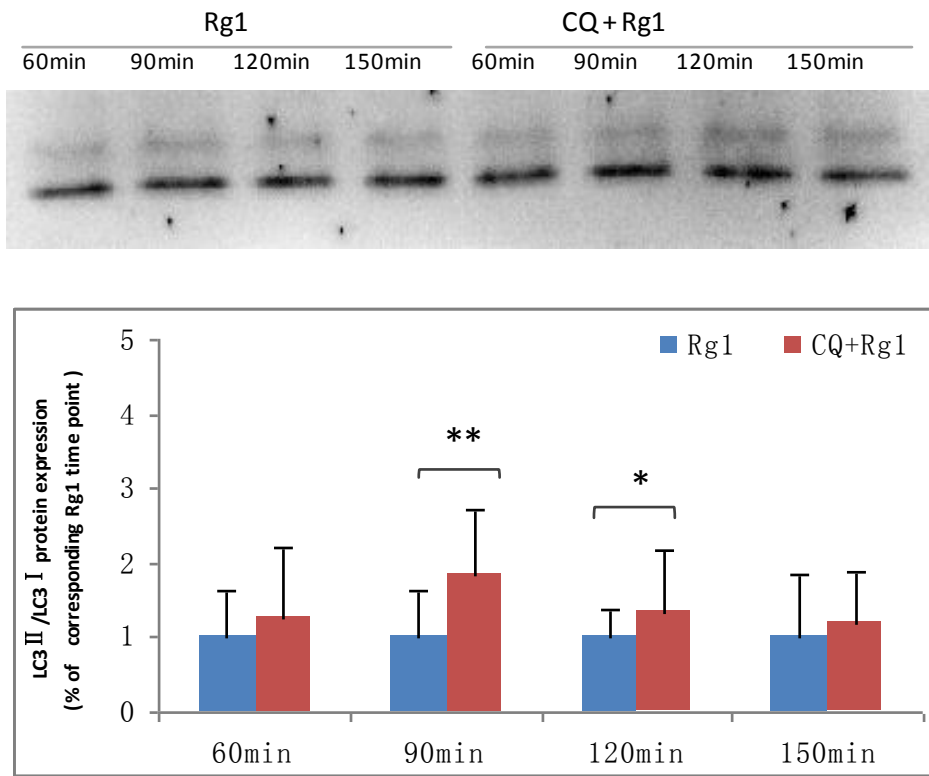

Fig. S4

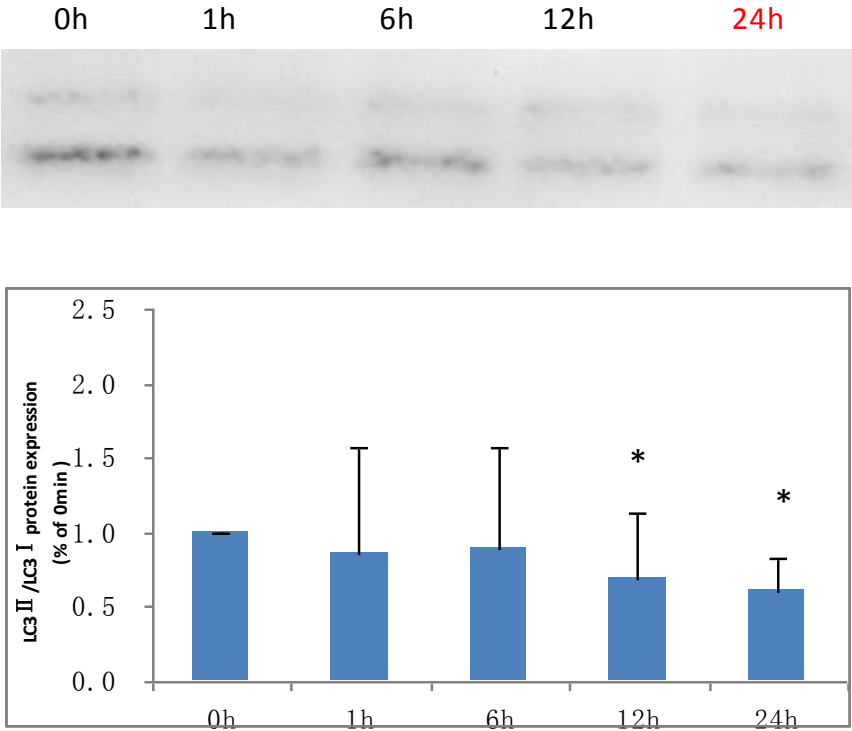

Fig. S5

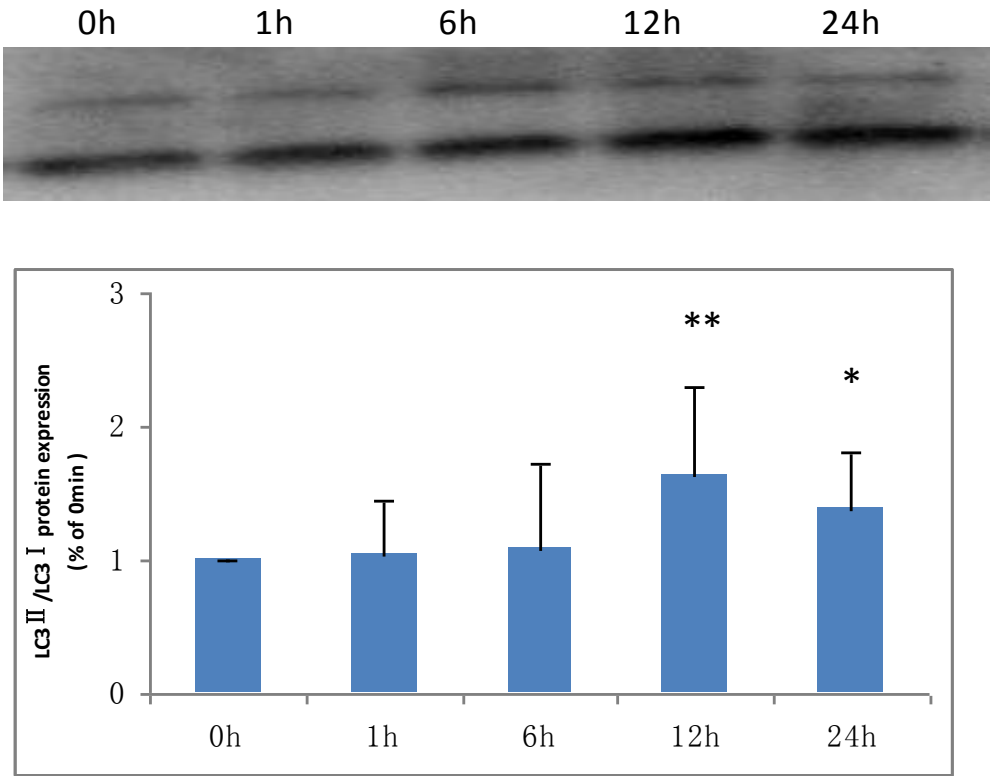

Supplement: Additional file 1: Figure S1. — Cell viability of H9c2 cells. Cell viability significantly decreased in a time-dependent manner between 30 and 240 min when compared with the control (0 min). Values are expressed as the mean ± SD, n = 3. ** p < 0.01, starvation model group verse control group. Figure S2. Effects of ginsenoside Rg1 on starvation-induced apoptosis in H9c2 cells using flow cytometric analysis. (PDF 346 kb) [file 12906_2016_1112_MOESM1_ESM.pdf]
